# Serum microRNA expression levels can predict lymph node metastasis in patients with early-stage cervical squamous cell carcinoma

**DOI:** 10.3892/ijmm.2013.1424

**Published:** 2013-06-21

**Authors:** JUNYING CHEN, DESHENG YAO, YUE LI, HONG CHEN, CHANJUAN HE, NAN DING, YAN LU, TINGYU OU, SHAN ZHAO, LI LI, FENGYI LONG

**Affiliations:** 1 Department of Gynecological Oncology, Affiliated Cancer Hospital of Guangxi Medical University, Nanning, Guangxi 530022, P.R. China; 2 Department of Gynecology, The First Affiliated Hospital of Guangxi Medical University, Nanning, Guangxi 530022, P.R. China; 3 Department of Health and Statistics, School of Public Health, Nanchang University, Nanchang, Jiangxi 330006, P.R. China

**Keywords:** cervical squamous cell carcinoma, microRNA, lymph node metastasis, serum marker

## Abstract

Circulating microRNA expression levels can serve as diagnostic/prognostic biomarkers in several types of malignant tumors; however, to our knowledge, there have been reports describing their value in cervical squamous cell carcinoma (SCC). In this study, we used hybridization arrays to compare the microRNA expression profiles in cervical squamous cell carcinomas (SCC) samples among patients with lymph node metastasis (LNM) or without LNM; 89 microRNAs were found to fit our inclusion criteria. Using quantitative PCR (qPCR), we examined the expression levels of these microRNAs in cervical cancer tissue, as well as in serum from patients and healthy women. We compared the expression levels between patients with LNM (n=40) and those without LNM (n=40) and healthy controls (n=20). Using regression analysis, we generated a comprehensive set of marker microRNAs and drew the fitted binormal receiver operating characteristic (ROC) curves to access the predictive value. We identified 6 serum microRNAs that can predict LNM in cervical SCC patients; these microRNAs were miR-1246, miR-20a, miR-2392, miR-3147, miR-3162-5p and miR-4484. The area under the curve (AUC) of the comprehensive set of serum microRNAs predicting LNM was 0.932 (sensitivity, 0.856; specificity, 0.850). The predictive value of the serum microRNAs was inferior to that in tissue (AUC 0.992; sensitivity, 0.967; specificity, 0.950; P=0.018). We compared the LNM predictive value of serum microRNAs and SCC antigen (SCC-Ag) by drawing fitted binormal ROC curves However, serum microRNA analysis is by far superior to serum SCC-Ag analysis (AUC 0.713; sensitivity, 0.612; specificity, 0.700; P<0.0001). Serum microRNAs are a good predictor of LNM with clinical value in early-stage cervical SCC.

## Introduction

Cervical carcinoma is the second most common type of cancer among women worldwide and results in approximately 300,000 deaths annually (1,2). This type of cancer is much more prevalent in developing countries, reflecting the success of screening programs on the incidence of cervical cancer in developed nations. Approximately 85% of pathological type cervical cancer cases are squamous cell carcinoma (SCC) (3). Although the International Federation of Gynecology and Obstetrics (FIGO) staging system for cervical carcinoma does not take into account the pelvic lymphadenopathy (LN) status, it is the most significant prognostic factor for patients with stages IB or IIA of the disease; the 5-year survival rate for patients declines dramatically from approximately 80–95% in patients without lymph node metastasis (LNM) to approximately 50–65% in patients with positive lymph nodes (3–5). Currently, there are no effective serum markers with the ability to predict LNM in cervical SCC patients. The FIGO staging system fails to detect LNM in approximately 15–20% of patients with early-stage cervical carcinoma (4). SCC antigen (SCC-Ag) is currently the most widely used SCC marker; it is a subfraction of TA-4 and a tumor-associated antigen belonging to the family of serine protease inhibitors. Serum levels of SCC-Ag have been found to correlate with tumor stage, tumor size, the amount of residual tumor remaining after treatment, recurrent or progressive disease, as well as survival in patients with SCC (6–10). However, SCC is not organ-specific (for cervix) or malignancy-specific. In apparently healthy women, the 99th percentile of circulating SCC-Ag is found at a level of 1.9 μg/l. Elevated levels have been found in patients with SCC of the vulva, vagina, head and neck, esophagus and lungs, as well as in patients with benign diseases of the skin (e.g., psoriasis, eczema), lungs (e.g., sarcoidosis), liver and kidneys (3,11,12). A normal initial SCC-Ag level cannot exclude the presence of LNM and extra-cervical spread, and hence is of limited use in treatment planning (13). These factors interfere with the specificity of serum SCC-Ag levels, particularly at relatively low threshold levels. According to our previous meta-analysis, the LNM predictive value of serum SCC-Ag levels in patients with cervical carcinoma was unable to provide satisfactory reference information for clinicians (sensitivity, 0.60; specificity, 0.76) (14).

MicroRNAs (also known as miRs) are small non-coding RNAs that are approximately 22 nt in size. They can modulate differentiation, growth, apoptosis and the proliferation of cells by regulating gene expression at the post-transcriptional level. MicroRNAs have crucial functions in human diseases, including cancer (15,16). A single microRNA can regulate hundreds of downstream genes, thus having a broad effect. As a result, information from microRNA profiling may provide better classification of cancer stages than the profiles of protein-coding genes. Due to their stability and presence in almost all body fluids, microRNAs constitute a novel class of non-invasive biomarkers (17–21). To date, however, to our knowledge, there are no reports describing the use of serum microRNA expression levels in patients with cervical SCC.

We hypothesized that some microRNAs found in tissue may be used as fingerprints to predict LNM in patients with early-stage cervical SCC (IB_1_-IIA_1_). Furthermore, their expression levels in serum may serve as non-invasive biomarkers. To address this hypothesis, we screened microRNAs in SCC tissue using hybridization arrays. We also performed quantitative RT-PCR (qRT-PCR) in a cohort of 100 paired samples (cervical tissue and paired serum from the same woman) to validate the predictive value of these microRNAs and their ability to serve as biomarkers.

## Materials and methods

### Research subjects and clinical samples

Between March 2010 and May 2012, we collected 100 cervical tissue samples and matched serum samples. In total, 80 patients had cervical SCC and 20 were healthy women. All patients were classified as FIGO stages IB_1_-IIA_1_ and had received radical hysterectomy and bilateral lymphadenectomy at the Department of Gynecological Oncology, Affiliated Cancer Hospital of Guangxi Medical University, Nanning, China. The healthy controls were women who had received regular health checks and did not possess any gynecological disease. Sample collections were performed prior to primary treatment. The study was approved by the Ethics Committee of Guangxi Cancer Prevention and Control Committee, and written informed consent was obtained from all participants. All tissue samples were immediately stored at −80°C after collection. Blood samples were obtained by venous puncture, clotted and centrifuged at room temperature. They were then also stored at −80°C. The personnel responsible for collecting the samples did not participate in patient grouping or experimental data analysis.

Each cervical tissue sample (approximately 300 mg) used for microRNA hybridization array analysis was a mixture from 3 different individuals (100 mg each). In total, 3 groups were analyzed: cervical SCC tissue from patients with LNM, cervical SCC tissue from patients without LNM and normal controls.

qRT-PCR was performed on 100 subjects, including 40 cervical SCC patients with LNM, 40 cervical SCC patients without LNM and 20 healthy women. There was no statistically significant difference in age among the patients in all 3 groups.

### MicroRNA profiling using hybridization arrays

The test operator and data analyst were blinded to the original source of samples and patient information. RNA isolation was completed using TRIzol reagent (Invitrogen/Life Technologies, Carlsbad, CA, USA). RNA quality and quantity were evaluated using the 260/280 ratio with a NanoDrop ND-1000 spectrophotometer (NanoDrop Technologies, Inc., Rockland, DE, USA) and an Agilent 2100 Bioanalyzer (Agilent Technologies, Palo Alto, CA, USA) (22). The microRNA expression profiles of the cervical SCC tissues were run on Human microRNA OneArray^®^ (Phalanx Biotech Group, Inc., Hsinchu, Taiwan). Probe design was based on the Sanger miRBase database, according to the characteristics of each sequence, corresponding to 1,450 mature microRNAs, and based on strict Tm values of the control and adjustment. For each chip, each microRNA sequence has 3 probes (23).

### Candidate microRNA confirmation and quantification

For each patient, we extracted small RNAs (<200 nt) from 50 mg cervical cancer tissue and 500 μl serum. Synthetic miR-238 from *C. elegans* was spiked into the serum samples prior to RNA extraction as an internal control as previously described (24). Small RNA was isolated using the miRcute microRNA isolation kit (DP501; Tiangen Biotech, Beijing, China). cDNA was obtained using the MiraMas™ kit (no. 5208; Bioo Scientific, Austin, TX, USA), using RNA Ligase to add the same adapter sequence to the 3′ ends of the small RNAs, followed by reverse transcription with M-MLV Reverse Transcriptase (Invitrogen/Life Technologies) to convert the small RNAs to cDNA templates for qPCR. The qPCR reaction was performed with diluted cDNA, (For + Rev) primer mixture, and SYBR Premix Ex Taq™ II (DRR081A; Takara Biotechnology Co., Dalian, China). The reverse primer was universal (5′-GTCCTTGGTGCCCGAGTG-3′).

We surveyed the literature and selected U6 as the endogenous control in cervical tissue, *C. elegans* miR-238 was used as an endogenous control for serum as previously described (24). The control sample and the test sample were placed in parallel into each 96-well plate. The ratios of the microRNAs were calculated using the equation 2^−ΔΔCT^, in which ΔΔCT = cycle threshold (CT_patient_ − CT_endogenous control_ − ΔCT_control sample_), ΔCT_control sample_ is the difference between the CT_control sample_ in one reaction and the mean of CT_control sample_ in all reactions (25). The coefficient of variation (CV) of CT values for the control sample between different plates for different patients was small and comparable (CV, 0.046 for miR-16; CV, 0.031 for miR-122). We selected the minimum microRNA expression sample and set the expression quantity of this sample as 1. The expression quantities of all the other samples were equal to the relative ratio.

### Statistical analysis

Statistical analysis was performed using Cluster and TreeView software (http://rana.lbl.gov/EisenSoftware.htm), SPSS software version 16.0 (SPSS Inc., Chicago, USA) and Rockit 1.1B2 software (University of Chicago, Chicago, IL, USA). All statistical tests were two-sided. P-values <0.05 were considered to indicate statistically significant differences. Due to the magnitude and range of the observed data, the results were log-transformed for analysis. For each microRNA, a receiver operating characteristic (ROC) curve was generated. Spearman correlation analysis was used to test the consistency of the tissue and serum expression levels in the same individual. Non-parametric tests were used to analyze the expression levels in the different groups. To increase the predictive accuracy of the serum microRNAs, multiple logistic regression analyses were performed to generate a comprehensive set of marker microRNAs. The fitted binormal ROC curve is displayed as the true positive rate (TPR) vs. the false positive rate (FPR).

## Results

### Phase I - Marker microRNA identification

The identification of marker microRNAs was carried out as previously described (26). First, we determined the expression levels of 1,450 microRNAs in cervical SCC and normal cervical tissue (total n=9) using hybridization arrays. MicroRNA expression was normalized to small nuclear RNA (RNU6B, RNU44 and RNU48), and the mean expression level was calculated. A total of 89 microRNAs were differentially expressed (P<0.05) at relatively high levels (intensity >1,000). Among these, 62 microRNAs were upregulated and 27 were downregulated. The microRNAs with high levels of expression in tissue were more easily detectable in serum. As a result, we selected these 89 microRNAs as our potential candidate markers for further research (Table I).

**Figure 1:**
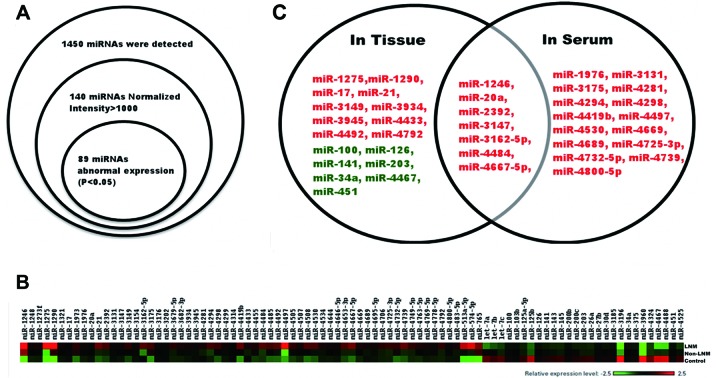
The screening process of the 7 candidate microRNAs. (A) According to the results of hybridization array (9 cases cervical tissue), we selected 89 microRNAs for further investigation. (B) Color-gram of the microRNA expression levels of the 89 microRNAs in the hybridization array samples. The 3 cervical samples from top to bottom were from patients with lymph node metastasis (LNM), patients without LNM and normal controls in order. (C) Based on the qPCR results of the 89 microRNAs (100 cases of tissue samples and 100 paired serum samples), we drew receiver operating characteristic (ROC) curves for each microRNA and selected the microRNA whose area under the ROC curve (AUC) was >70%. Seven microRNAs (miR-1246, -20a, -2392, -3147, -3162-5p, -4484 and -4667-5p) were selected for additional study and were found to have diagnostic value both in tissue and serum. Red, upregulated; green, downregulated.

**Figure 2:**
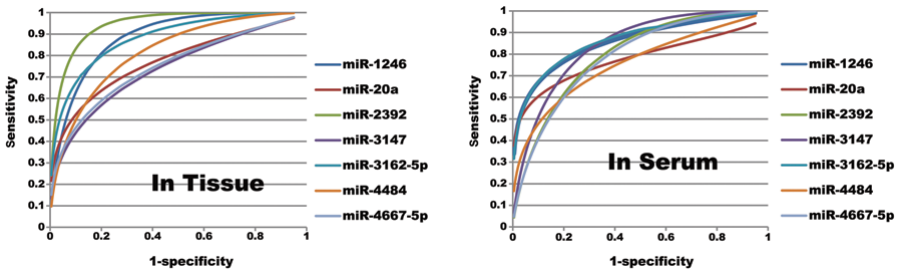
Receiver operating characteristic (ROC) curves of the 7 candidate microRNAs. All area under the ROC curve (AUC) values of these curves are >70%. The 7 candidate microRNAs have diagnostic value both in tissue and serum.

Subsequently, using qPCR, we measured the expression of these 89 microRNAs in both tissue and serum from 80 cervical SCC patients and 20 healthy women. Upon comparing patients with LNM (n=40) with those without LNM (n=40) and the healthy controls, we needed to control the isolation efficiency. Thus, tissue microRNA expression was normalized to U6, and serum microRNA was normalized to *C. elegans* miR-238, which was spiked into the serum prior to RNA isolation. We compared the microRNA expression levels in the different groups of patients and used the area under the ROC curve (AUC) >0.70 as our inclusion criteria. In SCC tissue, 17 microRNAs were upregulated and 7 were downregulated. In the serum from patients, 22 microRNAs were upregulated and all other microRNAs were expressed at similar levels. We followed-up on 7 candidate microRNAs for further investigation. These included miR-1246, miR-20a, miR-2392, miR-3147, miR-3162-5p, miR-4484 and miR-4667-5p. The criteria for further investigation of the 7 most promising candidates were: i) a high microRNA expression level in the cancer tissue of patients with LNM, ii) a high microRNA expression level in the serum of patients with LNM, and iii) quantification cycle values <35 to enable reliable detection (Figs. 1 and 2, and Table II).

### Phase II - Marker microRNA elimination

**Figure 3:**
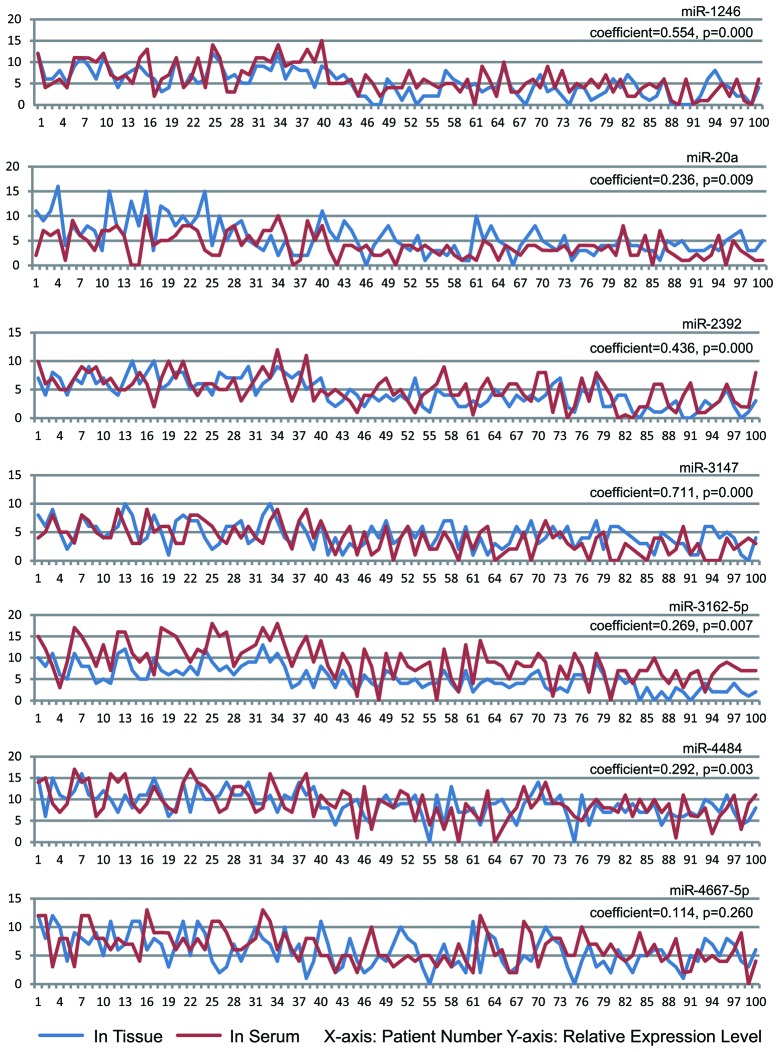
Expression levels of the 7 candidate microRNAs in tissue and serum. Spearman correlation analysis showed that, apart from miR4667-5p, the serum expression levels of all other microRNAs were consistent with the cancer tissue expression levels. Patient numbers 1–40 are patients with lymph node metastasis (LNM), numers 41–80 are patients without LNM and numbers 81–100 are the normal controls.

After identifying the candidate microRNAs in step one, we then eliminated the microRNAs which were susceptible to fluctuation in serum. As a relatively stable marker, serum expression should be consistent with the expression in cancer tissue, and minimal interference from other organs would be ideal. We performed spearman correlation analysis of the microRNA expression levels in tissue and paired serum samples (total n=100) in order to examine these 7 microRNAs as serum markers. We wanted to ensure that serum expression correlated with the expression in the cervical tissue. The results revealed that, apart from miR4667-5p, the other 6 microRNAs found in tissue and the paired serum sampels were positively correlated. Thus, the serum expression of these 6 microRNAs (miR-1246, miR-20a, miR-2392, miR-3147, miR-3162-5p and miR-4484) reflects the microRNA expression in the cervical tissue of the same subject (P<0.05). Although our results indicated that miR-4667-5p in the serum of cervical SCC patients may predict LNM, the serum expression was not consistent with the tissue expression (coefficient, 0.114; P=0.260). Thus, serum miR-4667-5p expression levels may be influenced by factors other than cervical cancer tissue. As a result, we ruled out this microRNA as a serum LNM marker in cervical SCC patients (Fig. 3).

**Figure 4:**
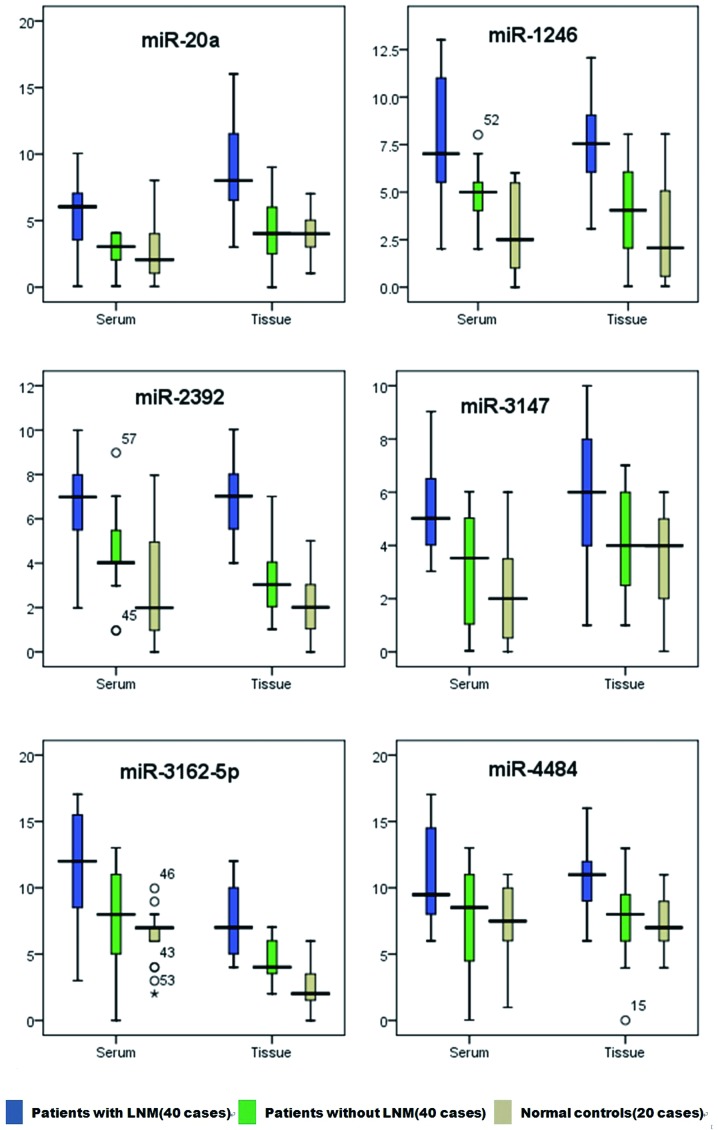
MicroRNA relative expression levels in patients with cervical carcinoma. All 6 candidate microRNA expression levels decreased in sequence from left to right [lymph node metastasis (LNM) cases > no LNM case > normal control]. The number displayed in the figure is the number of the sample.

To further refine our set of potential microRNA biomarkers, we ensured that the difference between patients with LNM and those without LNM should not be smaller than the difference between patients with LNM and the normal controls. In this manner, if the microRNA is a qualified LNM marker in cervical SCC patients, the expression in patients with LNM should be higher than the expression in patients without LNM and the expression in normal controls. Additionally, the expression in patients without LNM should not be lower than that in normal controls. According to this principle, using a non-parametric test, we compared the differences in microRNA expression levels between patients with LNM, those without LNM and the normal controls. The results revealed that 6 candidate microRNAs conformed to the inclusion criteria, and no microRNAs were eliminated (Fig. 4 and Table III).

### Phase III - Comprehensive predictive value of biomarker microRNAs

**Figure 5:**
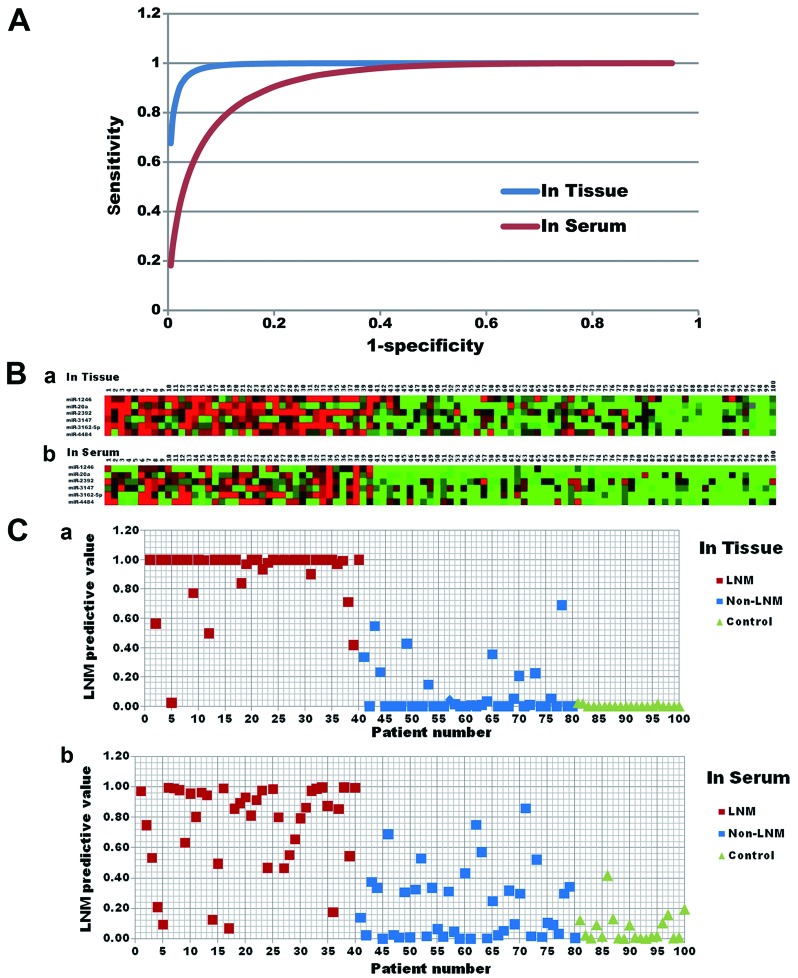
The lymph node metastasis (LNM) predictive ability of the comprehensive set of microRNAs. (A) The fitted binormal receiver operating characteristic (ROC) curves of the comprehensive set of microRNAs. The comprehensive set was made up of miR-1246, miR-20a, miR-2392, miR-3147, miR-3162-5p and miR-4484 and was generated through multiple regression analysis and integration. The ROC curves were drawn by Rockit software. In cancer tissue, the area under the ROC curve (AUC) was 0.992, and the maximum Youden index of this comprehensive set was 0.917 (sensitivity, 0.967; specificity, 0.950). In serum, the AUC was 0.932, and the maximum Youden index was 0.706 (sensitivity, 0.856; specificity, 0.850). (B) Color-gram of 6 candidate microRNAs in tissue and serum. (a) Marker microRNA expression levels in tissue. (b) Marker microRNA expression levels in serum. Numbers 1–40 were patients with LNM, numbers 41–80 were patients without LNM and numbers 81–100 were the normal controls. (C) Scatter diagram of the LNM predictive value of the comprehensive set of microRNAs. (a) LNM predictive value of the comprehensive set in tissue. (b) LNM predictive value of the comprehensive set in serum. Shown is the predictive value of the comprehensive set for each patient. The patients with higher values are those whose pelvic lymph nodes are more likely to be positive. Numbers 1–40 were patients with LNM, numbers 41–80 were patients without LNM and numbers 81–100 the were normal controls.

After confirmation and in order to access the comprehensive predictive value of biomarker microRNAs, we integrated these microRNAs (miR-1246, miR-20a, miR-2392, miR-3147, miR-3162-5p and miR-4484) into a comprehensive factor by regression analysis, and fitted binormal ROC curves by Rockit software. The results revealed that the comprehensive set of serum microRNA markers can predict LNM in cervical SCC patients with a sensitivity of 0.856, a specificity of 0.850 and at a threshold of 0.551 [AUC 0.932; 95% confidence interval (CI) 0.884–0.980]. However, this predictive value was inferior to that of the comprehensive set of tissue microRNA markers (sensitivity, 0.967; specificity, 0.950; at a threshold of 0.538; AUC 0.992; 95% CI 0.980–1.004). Overall, serum microRNAs are still reliable markers of LNM (Fig. 5).

**Figure 6:**
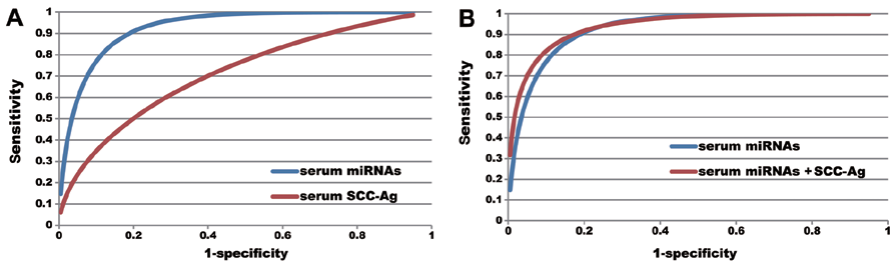
Comparison of the ability of serum microRNAs and squamous cell carcinoma antigen (SCC-Ag) to predict cervical cancer lymph node metastasis (LNM). (A) Comparison of the area under the ROC curve (AUC) of the fitted binormal receiver operating characteristic (ROC) curves between serum microRNA and SCC-Ag. The comprehensive set of microRNAs integrated with 6 microRNAs from serum had an AUC value of 0.932 and a maximum Youden index of 0.706 (sensitivity, 0.856; specificity, 0.850). AUC of serum SCC-Ag was 0.713, and the maximum Youden index was 0.312 (sensitivity, 0.612; specificity, 0.700). There was a statistically significant difference between the AUC values of the 2 ROC curves, P<0.0001. (B) Comparison of the AUC of the fitted binormal ROC curves between serum microRNA with SCC-Ag and serum microRNA only. Using regression analysis and integration, we added serum SCC-Ag into the LNM predictive set. While the AUC increased to 0.940, there was no statistically significant difference in comparison to the AUC of the serum microRNAs only, P=0.321.

We compared the LNM predictive value of serum microRNAs and SCC-Ag by drawing fitted binormal ROC curves. The AUC of serum SCC-Ag was 0.713 and the maximum Youden index was 0.312 (sensitivity, 0.612; specificity, 0.700). Comparing the AUC of the serum SCC-Ag and serum microRNA, the predictive accuracy of serum microRNA was by far superior to that of SCC-Ag (P<0.0001, Rockit software). In order to further increase the serum LNM predictive value, we integrated the serum SCC-Ag into the comprehensive set of serum microRNAs by regression analysis, and the revealed that although the AUC increased from 0.932 to 0.940, there was no statistically significant difference (P=0.321). Thus, the combination of serum SCC-Ag with serum microRNA levels does not increase the LNM predictive value in cervical SCC patients (Fig. 6).

## Discussion

To our knowledge, this is the first study of a comprehensive interrogation of circulating microRNA expression levels in cervical SCC patients with LNM. MicroRNAs are an abundant class of small, non protein-coding RNAs that function as negative regulators of gene expression. Recent research shows that certain microRNAs are associated with cancer and may correlate with tumor stage (27,28). Endogenous circulating microRNAs are stable, well protected from RNases, and remain stable even after being subjected to harsh conditions. Due to their stability and presence in almost all body fluids, microRNAs constitute a novel class of non-invasive biomarkers. A number of studies have described the usefulness of microRNAs as markers in a variety of diseases. For example, serum miR-21 levels have been described as a marker for necroinflammation in hepatitis C patients and miR-214 levels in the blood have been linked to breast cancer (29,30). Moreover, circulating microRNA levels have been specifically linked to LNM. For example, high serum miR-10b levels have been associated with LNM in lung cancer (31); miR-199a-3p levels in plasma have been associated with gastric cancer invasion and LNM stage (32); and serum microRNA levels have been shown to correlate with the tumor-node-metastasis stage in papillary thyroid carcinoma (33). However, to our knowledge, there are no studies concerning circulating microRNA expression levels and LNM in cervical SCC patients.

Currently, LNM in cervical SCC patients is identified by surgery, pathological analysis and imaging (34). However, these techniques have their limitations. SCC-Ag is the only marker which the National Academy of Clinical Biochemistry (NACB) recommends for cervical SCC patients. High SCC-Ag levels prior to treatment may indicate the presence of LNM or extra-cervical spread. Importantly, normal SCC-Ag levels do not exclude the presence of LNM (3,8–10,14). Other cervical cancer markers, such as carcinoembryonic antigen (CEA) and CA125, are primarily associated with cervical adenocarcinoma. Tissue polypeptide antigen (TPA) and tissue polypeptide-specific antigen (TPS) may have predictive value in cervical cancer; however, data concerning these 2 antigens are still conflicting and thus further investigation is required (3,35,36).

Pioneering studies on cervical carcinoma microRNAs have demonstrated that tissue microRNAs are potential tumor markers. miR-372 has been found to be downregulated in cervical carcinoma tissues compared with adjacent normal cervical tissues (37). miR-23b/uPA is involved in HPV-16 E6-associated cervical carcinoma development (38). The expression of miR-21 has been shown to increase with worsening clinical diagnosis (39). miR-34a suppresses cervical carcinoma invasion through the downregulation of Notch1 and Jagged1 (40). miR-20a promotes migration and invasion by regulating tankyrase, TRF1-interacting ankyrin-related ADP-ribose polymerase 2 (TNKS2) expression in human cervical carcinoma cells (41). The concordance of the microRNAs (miR-21, miR-20a and miR-34a) in our study with those from previous studies supports the validity of our findings in Phase I. Some tissue microRNAs, including miR-21 and miR-34a, were not good serum markers. This is possibly due to the fact that the cervix is not a vital metabolic or endocrine organ, and finding cervical lesions in serum is extremely difficult. There may also be other reasons for this: i) experimental errors from tissue RNA isolation, such as sample size errors or RNA degradation; ii) different normalization strategies in serum (*C. elegans* miR-238) and tissue (U6) may also contribute to the different numbers of unregulated microRNAs in tissue and serum.

The tumor specificity of a single microRNA may be limited by biological characteristics. However, microRNAs can regulate hundreds of downstream genes. Thus, the information obtained from microRNA profiling may provide a more accurate classification of cancer stages than the profiles obtained from protein-coding genes. In order to evaluate the predictive value of our comprehensive set of microRNAs, we integrated these marker microRNAs (miR-1246, miR-20a, miR-2392, miR-3147, miR-3162-5p and miR-4484) into a comprehensive set by regression analysis. The fitted binormal ROC curves showed that the AUC of the serum microRNAs was 0.932 (sensitivity, 0.856; specificity, 0.850). Although this predictive value was inferior to the value of these microRNAs in tissue (AUC 0.917; sensitivity, 0.967; specificity, 0.950), it was still superior to the predictive value of serum SCC-Ag (AUC 0.713; sensitivity, 0.612; specificity, 0.700) and is a good predictive marker of LNM in cervical SCC patients.

Before beginning the experiments, the study design was strictly reviewed. First, we screened the microRNAs in cervical tissue but not in serum in order to reduce the number of potentially interfering microRNAs from other organs. Second, each study participant had a clearly defined responsibility, including specimen collection and data analysis. The experimental operator did not participate in any of the experiments; thus, this is a double-blinded study. Third, in order to avoid repeated freezing and thawing of the RNA samples, we added the same adapter sequence to the 3′ ends of the small RNAs via reverse transcription, and all 89 microRNAs were subject to PCR in a single 96-well plate. Finally, we only selected patients with stage IB_1_-IIA_1_ SCC for our study in order to guarantee experimental accuracy. Lesions with a stage lower than stage IB_1_ are constantly mixed with surrounding tissue during specimen collection. Tumors with a stage higher than IIA_1_ usually receive neoadjuvant chemotherapy prior to radical surgery, and patients with these tumors were not included in this study.

Our results revealed that all microRNAs downregulated in cancer tissue were unable to predict LNM in the serum samples. This may be due to the fact that serum microRNAs originating from the cervix account for only a small portion of total serum microRNAs. We measured the microRNA expression levels in both tissue and serum. The results demonstrated that miR-1246, miR-20a, miR-2392, miR-3147, miR-3162-5p, miR-4484 and miR-4667-5p were all expressed in the serum, and that their expression levels in serum were consistent with their expression levels in cervical tissue. Unlike the others, the expression of miR-4667-5p in serum was not consistent with its expression in cervical tissue. As a result, we eliminated this microRNA from further analysis.

To our knowledge, our study is the first to describe the correlation between miR-1246 and cervical carcinoma. Pioneering studies suggest that miR-1246 is a novel target of p53, and that dual specificity tyrosine-phosphorylation-regulated kinase 1A (DYRK1A), a down syndrome-associated protein kinase, is a target of miR-1246. Thus, there may be a p53-miR-1246-DYRK1A-nuclear factor of activated T-cells (NFAT) pathway in cancer (42,43). Baek *et al*(44) reported that DYRK1A along with down syndrome candidate region 1 (DSCR1), which encodes a protein that suppresses vascular endothelial growth factor (VEGF)-mediated angiogenic signaling by the calcineurin pathway, may be sufficient to markedly diminish angiogenesis. On the other hand, Pigati *et al*(45) reported that the extracellular and cellular microRNA profiles in cancer tissue are different. For example, the bulk of miR-1246 produced by malignant mammary epithelial cells was released. However, the majority of these microRNAs produced by non-malignant mammary epithelial cells were retained. Therefore, microRNAs released into the serum do not necessarily reflect the abundance of microRNAs in the cell of origin. Recently, Baraniskin *et al*(46) reported that serum hsa-miR-1246 is likely a pseudo microRNA derived from U2 small nuclear RNA (snRNA) fragments (RNU2-1f). It is highly stable in serum and plasma and may serve as a novel diagnostic biomarker for pancreatic ductal adenocarcinoma and colorectal carcinoma patients for future prospective screening studies.

miR-20a, another marker microRNA identified in our study, has been more extensively investigated in terms of tumor biology. It is an important member of the miR-17-92 cluster, which is amplified and overexpressed in lung cancer, ovarian cancer and osteosarcoma (47-49). Several targets of miR-20 have been identified and include signal transducer and activator of transcription 3 (STAT3), BCL2/adenovirus E1B 19 kDa interacting protein 2 (BNIP2), connexin 43 (CX43), ephrin (EPH)B2, EPHB4, LRF and Unc-51-like kinase 1 (*C. elegans*) (ULK1) (50–55). Recently, Elkayam *et al*(56) reported that the structure of human argonaute 2 (Ago2) bound to miR-20a, at 2.2 Å resolution, is anchored at both ends by the Mid and PAZ domains and makes several kinks and turns along the binding groove. Thus, miR-20a binding confers remarkable stability on hAgo2, locking this otherwise flexible enzyme into a stable conformation. In cervical carcinoma, Kang *et al*(41) reported that miR-20a is overexpressed in cervical carcinoma-derived cell lines (HeLa and C-33A), where it enhances long-term cellular proliferation, migration and invasion. Consistent with these results, the inhibition of miR-20a suppressed these functions. Oncogenic TNKS2 is directly regulated by miR-20a, and the suppression of TNKS2 inhibits the colony formation, migration and invasion of cervical carcinoma cells. Taken together, these data demonstrate that miR-20a is a SCC-associated oncomir.

In addition to miR-1246 and miR-20a, this is the first clinical report describing 4 biomarker microRNAs (miR-2392, miR-3147, miR-3162-5p and miR-4484). Each of these 4 microRNAs was identified in 2010. While their exact biological function is unknown, deep sequencing of tumor tissue has revealed that they are expressed in cancer: miR-3162-5p and miR-3147 are found in melanoma (57,58), and miR-2392 and miR-4484 are expressed in malignant human B cells (59). In this study, we confirmed that they are associated with cervical carcinoma, which indicates that they should be further investigated as tumor-associated factors.

This had some limitations. While the presence of LNM is the most important prognostic factor associated with recurrent disease and poor survival, serum microRNA biomarkers of LNM cannot directly serve as prognostic factors of cervical SCC. Long-term follow-up studies are still required to confirm the correlation between serum microRNA levels and patient outcome. While our data is applicable to early-stage cervical SCC patients, it does not represent the condition of advanced cervical SCC patients. Additionally, all patients and healthy controls were from southern China. Thus, the results of this study may be influenced by local characteristics and may not represent the characteristics of patients of other races or who live in other areas.

Based on the data presented in this, as well as other studies, unique patterns of serum microRNAs may serve as non-invasive biomarkers for cancer development and prognosis. Therefore, using qRT-PCR to determine microRNA signatures in patients is a clinically applicable procedure. Due to the simplicity and reproducibility of obtaining a blood sample, biomarkers found in blood serum can be easily examined and may have great potential for cancer diagnosis. These serum markers, if validated in other populations and in prospective studies, may provide the opportunity for a randomized clinical trial to evaluate cervical SCC patients in which pelvic lymph nodes have been affected and who may have poor prognosis. It is important to expand the research scope, increase the number of subjects, and adjust the inclusion criteria appropriately in order to identify additional marker microRNAs and further increase the LNM prediction accuracy. In addition, due to non-invasive characteristics, serum microRNA signatures may be used to investigate the effects of surgery and chemo-radiotherapy by observing their dynamic expression levels and profiles.

In conclusion, the results from our study suggest that serum microRNAs may have great potential to serve as novel, non-invasive biomarkers for LNM in early-stage cervical SCC. miR-1246, miR-20a, miR-2392, miR-3147, miR-3162-5p and miR-4484 are potential biomarkers. Additional large-scale studies are required to fully explore the role of serum microRNAs in cervical SCC patients with LNM.

## Figures and Tables

**Table I:** Relative expression of the 89 microRNAs up- or downregulated in cervical sample A (cases with LNM).

Expression	MicroRNA^a^	Total
Upregulated	miR-1246, miR-1248, miR-1273f, miR-1275, miR-1290, miR-1321, miR-17, miR-1973, miR-1976, miR-20a, miR-21, miR-2392, miR-3131, miR-3147, miR-3149, miR-3154, miR-3162-5p, miR-3175, miR-3176, miR-3202, miR-3679-5p, miR-3682-3p, miR-3934, miR-3945, miR-4281, miR-4294, miR-4298, miR-4299, miR-4314, miR-4419b, miR-4433, miR-4455, miR-4484, miR-4485, miR-4492, miR-4497, miR-4505, miR-4507, miR-4508, miR-4530, miR-4534, miR-4644, miR-4646-5p, miR-4653-3p, miR-4667-5p, miR-4669, miR-4689, miR-4695-5p, miR-4706, miR-4725-3p, miR-4739, miR-4749-5p, miR-4763-5p, miR-4769-5p, miR-4778-5p, miR-4792, miR-4800-5p, miR-483-5p, miR-513a-5p, miR-574-5p, miR-765	62
Downregulated	Let-7a, Let-7b, Let-7c, miR-100, miR-103b, miR-125a-5p, miR-125b, miR-126, miR-141, miR-143, miR-145, miR-200b, miR-200c, miR-203, miR-26a, miR-27b, miR-30d, miR-3185, miR-3196, miR-34a, miR-375, miR-3960, miR-4324, miR-4467, miR-4488, miR-451, miR-4525	27

a Screen result by hybridization array, each microRNA relative intensity >1,000 at least, and expression abnormal in cervical sample A (cases with LNM).

LNM, lymph node metastasis.

**Table II:** Diagnostic value of microRNAs for LNM in patients with cervical SCC in cancer tissue and serum.

	In cancer tissue							In serum						
		
MicroRNA	AUC	95% CI	SE	Youden	Cut-off	Sen	Spe	AUC	95% CI	SE	Youden	Cut-off	Sen	Spe
miR-1246	0.881^a^	0.818–0.944	0.032	0.611	5.055	0.861	0.750	0.847^a^	0.767–0.927	0.041	0.575	6.010	0.715	0.860
miR-20a	0.761^a^	0.657–0.864	0.053	0.438	6.525	0.588	0.850	0.771^a^	0.664–0.878	0.055	0.504	5.050	0.604	0.900
miR-2392	0.945^a^	0.904–0.985	0.021	0.748	4.055	0.898	0.850	0.784^a^	0.695–0.872	0.045	0.444	5.000	0.744	0.700
miR-3147	0.722^a^	0.617–0.826	0.053	0.369	5.985	0.569	0.800	0.825^a^	0.748–0.903	0.040	0.521	3.975	0.821	0.700
miR-3162–5p	0.872^a^	0.805–0.940	0.035	0.597	5.775	0.797	0.800	0.869^a^	0.796–0.941	0.037	0.584	10.480	0.734	0.850
miR-4484	0.813^a^	0.727–0.899	0.044	0.477	9.390	0.727	0.750	0.73^a^	0.628–0.832	0.052	0.400	10.490	0.600	0.800
miR-4667–5p	0.737^a^	0.635–0.839	0.052	0.389	7.000	0.589	0.800	0.78^a^	0.689–0.872	0.047	0.427	6.020	0.727	0.700

Youden, maximum Youden index; Cut-off, sensitivity (Sen) and specificity (Spe) values were according to the maximum Youden index. LNM, lymph node metastasis; CI, confidence interval; AUC, area under the receiver operating characteristic (ROC) curve.

a P<0.001.

**Table III:** The expression level of each candidate microRNA in the different groups of tissue (or serum) samples.

MicroRNA	In tissue or serum	Patients with LNM		Patients without LNM		Control		P-value		
			
		Median	Interquartile range	Median	Interquartile range	Median	Interquartile range	A/B	A/C	B/C
miR-1246	Tissue	7.555	6.055–9.060	4.040	2.060–5.060	2.070	0.575–5.065	0.000	0.000	0.396
	Serum	10.000	6.000–11.000	5.000	3.990–6.000	2.505	1.010–5.485	0.000	0.000	0.005
miR-20a	Tissue	8.010	4.525–10.515	4.025	3.010–5.995	4.015	3.025–5.030	0.000	0.001	0.747
	Serum	6.060	3.565–7.075	3.070	2.070–4.060	2.065	1.050–4.020	0.000	0.016	0.001
miR-2392	Tissue	7.025	6.015–8.015	3.040	2.040–4.050	2.010	1.040–3.030	0.000	0.000	0.001
	Serum	6.010	5.005–7.500	4.020	3.475–5.990	1.985	0.970–4.955	0.000	0.000	0.005
miR-3147	Tissue	6.010	3.990–7.010	3.990	2.500–5.985	3.990	2.005–5.985	0.002	0.001	0.460
	Serum	5.025	3.990–7.025	3.030	1.040–5.025	2.005	0.520–3.495	0.000	0.000	0.038
miR-3162–5p	Tissue	8.010	6.000–9.005	4.020	3.010–6.015	2.025	1.520–3.505	0.000	0.000	0.000
	Serum	12.015	9.01–15.010	7.995	5.020–10.015	6.990	5.985–7.005	0.000	0.000	0.005
miR-4484	Tissue	10.990	9.500–12.500	8.490	6.000–9.500	5.995	7.000–8.980	0.000	0.000	0.268
	Serum	10.980	7.990–13.990	8.020	5.505–10.010	7.490	6.010–9.985	0.001	0.000	0.423

LNM, lymph node metastasis. A/B: Group A compared with B; A/C: Group A compared with C; B/C: Group B compared with C.

## References

[1] Hu X, Schwarz JK, Lewis JS (2010). A microRNA expression signature for cervical cancer prognosis. Cancer Res.

[2] Jemal A, Bray F, Center MM, Ferlay J, Ward E, Forman D (2011). Global cancer statistics. CA Cancer J Clin.

[3] Sturgeon CM, Duffy MJ, Hofmann BR (2010). National Academy of Clinical Biochemistry Laboratory Medicine Practice Guidelines for use of tumor markers in liver, bladder, cervical, and gastric cancers. Clin Chem.

[4] Hacker NF, Berek JS, Hacker NF (2000). Cervical cancer. Practical Gynecologic Oncology.

[5] (2003). Neoadjuvant chemotherapy for locally advanced cervical cancer: a systematic review and meta-analysis of individual patient data from 21 randomised trials. Eur J Cancer.

[6] Lee YY, Choi CH, Sung CO (2012). Prognostic value of pre-treatment circulating monocyte count in patients with cervical cancer: comparison with SCC-Ag level. Gynecol Oncol.

[7] Huang EY, Wang CJ, Chen HC (2008). Multivariate analysis of para-aortic lymph node recurrence after definitive radiotherapy for stage IB-IVA squamous cell carcinoma of uterine cervix. Int J Radiat Oncol Biol Phys.

[8] Ogino I, Nakayama H, Okamoto N, Kitamura T, Inoue T (2006). The role of pretreatment squamous cell carcinoma antigen level in locally advanced squamous cell carcinoma of the uterine cervix treated by radiotherapy. Int J Gynecol Cancer.

[9] Nakamura K, Okumura Y, Kodama J, Hongo A, Kanazawa S, Hiramatsu Y (2010). The predictive value of measurement of SUVmax and SCC-antigen in patients with pretreatment of primary squamous cell carcinoma of cervix. Gynecol Oncol.

[10] van de Lande J, Davelaar EM, von Mensdorff-Pouilly S (2009). SCC-Ag, lymph node metastases and sentinel node procedure in early stage squamous cell cervical cancer. Gynecol Oncol.

[11] Matsuda H, Mori M, Tsujitani S, Ohno S, Kuwano H, Sugimachi K (1990). Immunohistochemical evaluation of squamous cell carcinoma antigen and S-100 protein-positive cells in human malignant esophageal tissues. Cancer.

[12] Krzystek-Korpacka M, Matusiewicz M, Diakowska D, Grabowski K, Blachut K, Banas T (2007). Upregulation of VEGF-C secreted by cancer cells and not VEGF-A correlates with clinical evaluation of lymph node metastasis in esophageal squamous cell carcinoma (ESCC). Cancer Lett.

[13] Gaarenstroom KN, Bonfrer JM, Korse CM, Kenter GG, Kenemans P (1997). Value of Cyfra 21-1, TPA, and SCC-Ag in predicting extracervical disease and prognosis in cervical cancer. Anticancer Res.

[14] Chen J, Yao D, Wu Z (2012). Diagnostic value of serum squamous cell carcinoma antigen on lymphatic metastasis in early-stage cervical cancer: a meta-analysis. Cancer Res Prev Treat.

[15] Enfield KS, Stewart GL, Pikor LA (2011). MicroRNA gene dosage alterations and drug response in lung cancer. J Biomed Biotechnol.

[16] Medina PP, Nolde M, Slack FJ (2010). OncomiR addiction in an in vivo model of microRNA-21-induced pre-B-cell lymphoma. Nature.

[17] Wang XW, Heegaard NH, Orum H (2012). MicroRNAs in liver disease. Gastroenterology.

[18] Liu R, Chen X, Du Y (2012). Serum microRNA expression profile as a biomarker in the diagnosis and prognosis of pancreatic cancer. Clin Chem.

[19] Jurmeister S, Baumann M, Balwierz A (2012). MicroRNA-200c represses migration and invasion of breast cancer cells by targeting actin-regulatory proteins FHOD1 and PPM1F. Mol Cell Biol.

[20] Hennessey PT, Sanford T, Choudhary A (2012). Serum microRNA biomarkers for detection of non-small cell lung cancer. PLoS One.

[21] Wu Q, Lu Z, Li H, Lu J, Guo L, Ge Q (2011). Next-generation sequencing of microRNAs for breast cancer detection. J Biomed Biotechnol.

[22] Santos CF, Kurhanewicz J, Tabatabai ZL (2010). Metabolic, pathologic, and genetic analysis of prostate tissues: quantitative evaluation of histopathologic and mRNA integrity after HR-MAS spectroscopy. NMR Biomed.

[23] Hsieh CH, Rau CS, Jeng JC (2012). Whole blood-derived microRNA signatures in mice exposed to lipopolysaccharides. J Biomed Sci.

[24] Cermelli S, Ruggieri A, Marrero JA, Ioannou GN, Beretta L (2011). Circulating microRNAs in patients with chronic hepatitis C and non-alcoholic fatty liver disease. PLoS One.

[25] Hu Z, Chen X, Zhao Y (2010). Serum microRNA signatures identified in a genome-wide serum microRNA expression profiling predict survival of non-small-cell lung cancer. J Clin Oncol.

[26] Wulfken LM, Moritz R, Ohlmann C (2011). MicroRNAs in renal cell carcinoma: diagnostic implications of serum miR-1233 levels. PLoS One.

[27] Iorio MV, Ferracin M, Liu CG (2005). MicroRNA gene expression deregulation in human breast cancer. Cancer Res.

[28] Jamieson NB, Morran DC, Morton JP (2012). MicroRNA molecular profiles associated with diagnosis, clinicopathologic criteria, and overall survival in patients with resectable pancreatic ductal adenocarcinoma. Clin Cancer Res.

[29] Bihrer V, Waidmann O, Friedrich-Rust M (2011). Serum microRNA-21 as marker for necroinflammation in hepatitis C patients with and without hepatocellular carcinoma. PLoS One.

[30] Schwarzenbach H, Milde-Langosch K, Steinbach B, Muller V, Pantel K (2012). Diagnostic potential of PTEN-targeting miR-214 in the blood of breast cancer patients. Breast Cancer Res Treat.

[31] Roth C, Kasimir-Bauer S, Pantel K, Schwarzenbach H (2011). Screening for circulating nucleic acids and caspase activity in the peripheral blood as potential diagnostic tools in lung cancer. Mol Oncol.

[32] Li C, Li JF, Cai Q (2012). MiRNA-199a-3p in plasma as a potential diagnostic biomarker for gastric cancer. Ann Surg Oncol.

[33] Yu S, Liu Y, Wang J (2012). Circulating microRNA profiles as potential biomarkers for diagnosis of papillary thyroid carcinoma. J Clin Endocrinol Metab.

[34] Sakuragi N (2007). Up-to-date management of lymph node metastasis and the role of tailored lymphadenectomy in cervical cancer. Int J Clin Oncol.

[35] Juang CM, Wang PH, Yen MS, Lai CR, Ng HT, Yuan CC (2000). Application of tumor markers CEA, TPA, and SCC-Ag in patients with low-risk FIGO stage IB and IIA squamous cell carcinoma of the uterine cervix. Gynecol Oncol.

[36] Gaarenstroom KN, Kenter GG, Bonfrer JM (2000). Can initial serum cyfra 21-1, SCC antigen, and TPA levels in squamous cell cervical cancer predict lymph node metastases or prognosis?. Gynecol Oncol.

[37] Tian RQ, Wang XH, Hou LJ (2011). MicroRNA-372 is down-regulated and targets cyclin-dependent kinase 2 (CDK2) and cyclin A1 in human cervical cancer, which may contribute to tumorigenesis. J Biol Chem.

[38] Au Yeung CL, Tsang TY, Yau PL, Kwok TT (2011). Human papillomavirus type 16 E6 induces cervical cancer cell migration through the p53/microRNA-23b/urokinase-type plasminogen activator pathway. Oncogene.

[39] Deftereos G, Corrie SR, Feng Q (2011). Expression of mir-21 and mir-143 in cervical specimens ranging from histologically normal through to invasive cervical cancer. PLoS One.

[40] Pang RT, Leung CO, Ye TM (2010). MicroRNA-34a suppresses invasion through downregulation of Notch1 and Jagged1 in cervical carcinoma and choriocarcinoma cells. Carcinogenesis.

[41] Kang HW, Wang F, Wei Q (2012). miR-20a promotes migration and invasion by regulating TNKS2 in human cervical cancer cells. FEBS Lett.

[42] Zhang Y, Liao JM, Zeng SX, Lu H (2011). p53 downregulates Down syndrome-associated DYRK1A through miR-1246. EMBO Rep.

[43] Liao JM, Zhou X, Zhang Y, Lu H (2012). MiR-1246: a new link of the p53 family with cancer and Down syndrome. Cell Cycle.

[44] Baek KH, Zaslavsky A, Lynch RC (2009). Down’s syndrome suppression of tumour growth and the role of the calcineurin inhibitor DSCR1. Nature.

[45] Pigati L, Yaddanapudi SC, Iyengar R (2010). Selective release of microRNA species from normal and malignant mammary epithelial cells. PLoS One.

[46] Baraniskin A, Nopel-Dunnebacke S, Ahrens M (2013). Circulating U2 small nuclear RNA fragments as a novel diagnostic biomarker for pancreatic and colorectal adenocarcinoma. Int J Cancer.

[47] Matsubara H, Takeuchi T, Nishikawa E (2007). Apoptosis induction by antisense oligonucleotides against miR-17-5p and miR-20a in lung cancers overexpressing miR-17-92. Oncogene.

[48] Huang G, Nishimoto K, Zhou Z, Hughes D, Kleinerman ES (2012). miR-20a encoded by the miR-17-92 cluster increases the metastatic potential of osteosarcoma cells by regulating Fas expression. Cancer Res.

[49] Fan X, Liu Y, Jiang J (2010). miR-20a promotes proliferation and invasion by targeting APP in human ovarian cancer cells. Acta Biochim Biophys Sin (Shanghai).

[50] Chai H, Liu M, Tian R, Li X, Tang H (2011). miR-20a targets BNIP2 and contributes chemotherapeutic resistance in colorectal adenocarcinoma SW480 and SW620 cell lines. Acta Biochim Biophys Sin (Shanghai).

[51] Zhang M, Liu Q, Mi S (2011). Both miR-17-5p and miR-20a alleviate suppressive potential of myeloid-derived suppressor cells by modulating STAT3 expression. J Immunol.

[52] Li X, Pan JH, Song B (2012). Suppression of CX43 expression by miR-20a in the progression of human prostate cancer. Cancer Biol Ther.

[53] Wang W, Feng L, Zhang H (2012). Preeclampsia up-regulates angiogenesis-associated microRNA (i.e., miR-17, -20a, and -20b) that target ephrin-B2 and EPHB4 in human placenta. J Clin Endocrinol Metab.

[54] Poliseno L, Pitto L, Simili M (2008). The proto-oncogene LRF is under post-transcriptional control of MiR-20a: implications for senescence. PLoS One.

[55] Wu H, Wang F, Hu S (2012). MiR-20a and miR-106b negatively regulate autophagy induced by leucine deprivation via suppression of ULK1 expression in C2C12 myoblasts. Cell Signal.

[56] Elkayam E, Kuhn CD, Tocilj A (2012). The structure of human argonaute-2 in complex with miR-20a. Cell.

[57] Stark MS, Tyagi S, Nancarrow DJ (2010). Characterization of the melanoma miRNAome by deep sequencing. PLoS One.

[58] Persson H, Kvist A, Rego N (2011). Identification of new microRNAs in paired normal and tumor breast tissue suggests a dual role for the ERBB2/Her2 gene. Cancer Res.

[59] Jima DD, Zhang J, Jacobs C (2010). Deep sequencing of the small RNA transcriptome of normal and malignant human B cells identifies hundreds of novel microRNAs. Blood.

